# Tobacco—A Parable

**Published:** 1881-10

**Authors:** 


### TOBACCO—A PARABLE.

Then shall the kingdom of Satan be likened to a grain of tobacco-seed; which, though exceedingly small, being cast into the ground, grew, and became a great plant, and spread its leaves rank and broad, so that huge and vile worms formed a habitation thereon. And it came to pass, in the course of time, that the sons of men looked upon it, and thought it beautiful to look upon, and much to be desired to make lads look big and manly. So they put forth their hands and did chew thereof. And some it made sick, and others to vomit most filthily. And it further came to pass that those who chewed it became weak and unmanly, and said "we are enslaved, and can't cease from chewing it." And the mouths of all that were enslaved became foul; and they were seized with a violent spitting; and they did spit, even in ladies' parlors, and in the house of the Lord of Hosts. And the saints of the Most High were greatly plagued thereby. And in the course of time it came also to pass that others snuffed it; and they were taken suddenly with fits, and they did sneeze with a great and mighty sneeze, insomuch that their eyes filled with tears, and they did look exceedingly silly. And yet others cunningly wrought the leaves thereof into rolls, and did set fire to one end thereof, and did suck vehemently at the other end thereof, and did look very grave and calf-like; and the smoke of their torment ascended up forever and forever. And the cultivation thereof became a great and mighty business in the earth, and the merchantmen waxed rich by the commerce thereof. And it came to pass that the saints of the Most High defiled themselves therewith; even the poor, who could not buy shoes, nor bread, nor books for the little ones, spent their money for it. And the Lord was greatly displeased therewith, and said: "Wherefore this waste; and why do these little ones lack bread and shoes and books? Turn now your fields into corn and wheat; and put this evil thing far from you; and be separate, and defile not yourselves any more; and I will bless you and cause my face to shine on you." But with one accord they all exclaimed: "We cannot cease from chewing, snuffing, and puffing—we are slaves."—*Christian Secretary.*

